# Sexual Dysfunction in Women with Type 2 Diabetes Mellitus

**Published:** 2015-05

**Authors:** Forouzan Elyasi, Zahra Kashi, Bentolhoda Tasfieh, Adele Bahar, Mohammad Khademloo

**Affiliations:** 1 Psychiatry and Behavioral Sciences Research Center, Mazandaran University of Medical Science, Sari, Iran;; 2 Diabetes Research Center, Mazandaran University of Medical Sciences, Sari, Iran;; 3 General Physician, Mazandaran University of Medical Sciences, Sari, Iran;; 4 Diabetes Research Center, Mazandaran University of Medical Sciences, Sari, Iran;; 5 Department Community Medicine, Mazandaran University of Medical Sciences, Sari, Iran

**Keywords:** Diabetes Melitus, Sexual dysfunctions, Psychological, Female

## Abstract

**Background:**

Sexual dysfunction (SD) is one of the important problems in diabetic patients.

The present study aimed to determine the prevalence of sexual problems in Iranian women with type 2 diabetes mellitus.

**Methods:**

A cross-sectional study was conducted among type 2 diabetic women who visited two outpatient endocrine clinics, namely Imam Hospital and Tuba clinic (Sari, Iran) in 2012. Patients were asked to complete two validated questionnaires: Female Sexual Function Index (FSFI) and The Hospital Anxiety and Depression Scale (HADS) as well as a demographic questionnaire. Analysis was performed using descriptive and analytical tests. P<0.05 was considered to be significant.

**Results:**

One hundred and fifty women with type 2 diabetes were investigated. Most of the cases aged 40-44 years old. The mean of the total score of the FSFI questionnaire was 22. The prevalence of sexual dysfunction was 78.7% (CI: 71.4-84.4); among these, 58% (CI: 50.0-65.6) reported problems in lubrication, 50% (CI: 42.1-57.9) complained of decreased sexual desire, 50% (CI: 42.1-57.9) had problems with arousal, 47.3% (CI: 39.5-55.3) had dyspareunia, 32.7% (CI: 25.7-40.5) complained of orgasmic dysfunction and 42.7% (CI: 35.0-50.7) reported problems in sexual satisfaction.

With regard to the results of the HADS questionnaire, 58.7% (CI: 50.7-66.2) of the patients had depression and 96.7% (CI: 92.4-98.6) had anxiety.

**Conclusion:**

This study showed the high prevalence of sexual dysfunction in diabetic women, especially among those complaining of depression. Health care professionals dealing with diabetic patients should be aware of possible presence of sexual dysfunction in female patients.

## Introduction

About 285 million people are affected with diabetes mellitus (DM) in the world and by the year 2030, 439 million people are expected to suffer from the disease. In addition, by 2025 the largest increase in DM prevalence will occur in the developing countries.^1^ DM is known to cause different medical, psychological and sexual complications.^2^ Sexual dysfunction (SD) can also be an early sign of DM.^3^ The causes of SD in women can be divided into psychological and organic etiologies. Among non-gynecological organic etiologies, hormonal abnormalities, autonomic neuropathies (parasympathetic nervous system releases endothelial nitric oxide synthase, ENOS) as complications of DM, along with vascular insufficiency due to atherosclerosis are of most importance.^3^^,^^4^ It seems that somatic sensory system is affected by DM and introits vagina, labia minor and clitoris are the most deteriorated parts of genitalia in diabetic women. Although sexual complications are not present in all patients with DM, medications can improve blood flow in clitoris.^5^^,^^6^ It is argued that neuropathies, vascular impairments and psychological discomforts are the most recognized factors among the etiologies of SD in diabetic women.^7^

The prevalence of SD in diabetic women is estimated to be 20-80%.^8^ In the 1950s, SD in diabetic men caught attention, but SD in diabetic women remained completely neglected until Kolodny presented his article in 1971.^9^ Despite over 70 years of investigations in the field of DM in women, it has still remained a controversial issue.^10^ Sexual disorders have been studied extensively in men with DM,^11^^,^^12^ but the sexual problems in diabetic women has only recently received attention^2^^,^^5^^,^^13^ and some contradictory results have been presented.^14^ Sexual problems in both men and women with DM deserve further researches. Causes of SD are numerous and the neuroendocrinological background is complex.^3^ Research on DM and female SD in diabetic women is not only scarring, but also suffer from methodological problems such as small sample size, absence of control group and lack of differentiating between different types of DM, presence of complications, psychosocial adjustments to the disease, quality of marital relationship and depressive disorders.^2^ Despite over 70 years of research on the sexuality of females with diabetes, mellitus remains a controversial issue. There is a debate that the type of diabetes has an impact on the emergence of SD in women with diabetes.^15^ Islam, the religion of the vast majority of Iranians, has logically welcomed the positive attitudes towards legal sexual relationships in the context of marriage. However, social attitudes to the subject of sexual function are still taboo in many families and most people in Iran avoid talking about sexual function. There is a limited data regarding sexual dysfunction prevalence in patients with diabetes for both genders in Iran.^15^

Sexual health is an important, but often neglected component of health care in diabetic patients.^16^ There are few studies about SD in women with DM type 2 in Iran.^15^^,^^16^ The present study aimed to (i) determine the prevalence of sexual problems in women with DM type 2, (ii) investigate the influences of DM-related factors on female sexuality, and (iii) assess the influences of depression and anxiety on sexual function.

## Patients and Methods

A cross-sectional study was conducted among 150 females with type 2 DM who visited two outpatient endocrine clinics of Imam Hospital and Tuba clinic (Sari, Iran) during 2012. The sample size was estimated based on a single proportion design. A study with a sample of 150 diabetic patients would have a power of 80 % to detect a difference of 5% (45-55%) at a significant level of 0.05. The actual sample size obtained for this study was 150 women with type 2 diabetes. The samples were selected in a consecutive procedure from January 2012 to March 2012. The patients were defined to be eligible for inclusion if they were female, had type 2 diabetes, were 18-65 years old, did not have any other health problems except for controlled hypothyroidism and complications of DM, were married for at least 1 year, and have had a stable marital relationship. The exclusion criteria were records of mastectomy, bilateral hystero-oophorectomy, pregnancy, presence of ongoing sexual disorder in patient’s spouse, presence of sexual disorders before developing DM, and taking psychotropic drugs except for benzodiazepines.

Patients were asked to complete three validated questionnaires, namely Female Sexual Function Index (FSFI), the Hospital Anxiety and Depression Scale (HADS) and a demographic questionnaire at the clinics when they were on a waiting list. Privacy and confidentiality were assured.

The medical records of the patients were used to obtain data on the duration of DM, use of medications (including antihypertensive medications, oral hypoglycemic agents, insulin), body mass index (BMI), HbA1c, microvascular complications (such as neuropathy, retinopathy and nephropathy), macrovascular complications (such as hypertension, coronary artery disease), hyperlipidemia, history of cerebrovascular attack and previous myocardial infarction. Retinopathy was defined as having proliferative retinopathy in the past or at the time of the study assessed by a full examination of fundus performed by an ophthalmologist. Nephropathy was defined as a positive history of microalbuminuria, macroalbuminuria, or based on positive results of 24-hour urine test. The values of glycosylated hemoglobin (HbA1c) were obtained from their medical records of their last clinic visits. This topic included recording each patient’s age, educational status, occupation, income, number of children, homeownership, history of psychiatrist visits and history of any psychotropic drug consumption.

*Female Sexual Function Index (FSFI)*

Sexual function was measured in the women by using a standard questionnaire. The female sexual index is a known instrument assessing sexual function in women using 6 domains and 19 items. Individual items were assigned to six domains of female sexual functions: desires, arousal, lubrication, orgasm, satisfaction and pain during sexual intercourse.^17^ Scores of the six domains were added to obtain the total scale scores. For individual domain scores, scores of the individual items that compromise the domain were multiplied by the domain factors, where a higher score indicated lower sexual function.^17^ The overall test-retest reliability coefficients in the original form of the questionnaire were high for each form of the individual domains (r=0.79 to r=0.86). Cronbach’s alpha values for internal consistency were high (≥0.82). A good construct validity was demonstrated.^17^ FSFI is available in many languages and multiple researches in different countries such as Finland, Japan, Egypt, Malaysia and Iran support reliability and psychometric validity of the questionnaire in the assessment of key dimensions of female sexual function.^18-21^ Most of these studies have supported the use of the FSFI for assessing sexual function, not only in clinical samples but also in general populations. The Iranian version of the FSFI is a validated and locally accepted questionnaire for the assessment of female sexual function. This questionnaire had good psychometric properties in two separate researches.^21^^,^^22^ The overall test-retest reliability coefficients were high for each domain (r=0.73 to 0.86) and the range of internal consistency was acceptable (α=0.72 to α=0.90).^21^ In another study in Iran, the FSFI was a valid and reliable instrument to measure multidimensional aspects of female SD.^22^ The Iranian version of FSFI showed an excellent overall performance (area under the curve (AUC)=0.917).^22^ A total score of 28 was considered as the optimal cut off point for the Iranian version of the FSFI to distinguish between women with SD and those with normal sexual function (sensitivity 83%, specificity 82%). Higher scores of the FSFI indicate fewer problems in sexual function and lower scores demonstrate more problems. There were also other cut off points for the different subscales of the questionnaire: 3.3 for desire, 3.4 for arousal, Lubrication, and orgasm, and 3.8 for satisfaction and pain.^22^

*Hospital Anxiety and Depression Scale (HADS)*

The Hospital Anxiety and Depression Scale is a brief, widely used screening tool to measure psychological distress. It is sensitive to changes both during the course of illness and in response to medical and psychological management.^23^ The HADS contains 14 items and consists of two subscales, anxiety and depression; each item is rated based on a four point scale. The maximum score for both anxiety and depression subscales is 21. Scores 0-7, on either subscale are considered to be “normal”, while scores 11 or more represent a significant psychological morbidity, and scores 8-10 indicates a “borderline” status.^24^

The preliminary validation study of the Iranian version of HADS has demonstrated it to be a reliable and valid instrument with good psychometric properties for the measurement of psychological distress among patients with cancer and Iranian clinical population.^25^^,^^26^

Reliability was tested using Cronbach’s Coefficient Alpha and it was found to be 0.86 for the HADS depression subscale and 0.78 for the anxiety subscale.^25^ Also, different studies about HADS demonstrated that the HADS was a reliable and valid instrument in Iranian clinical population.

The Ethics Committee of Mazandaran University of Medical Sciences approved the study. All patients were entered into the study after giving an informed verbal consent. Diabetic women were informed by female interviewers and were assured of the confidentiality of the data. 

Analysis was performed by SPSS statistical software. Frequencies and its 95% CI, OR and its 95% CI were reported and student t-test and X2 were used to detect the association between demographic and clinical characteristics of the patients. The level of significance used was considered to be P<0.05. 

## Results

*Descriptive*

One hundred and fifty women with type 2 diabetes mellitus (DM) were studied. Most cases were 40-44 years of age. The mean age of participants was 42±10.1 years, the mean duration of DM was 7.57±5.5 years, the mean of the body mass index (BMI) was 30.87±4.52, and the mean of the last HbA1c was 7.90±1.75. The demographic and clinical characteristics of the study samples are shown in table 1.

**Table 1:** The demographic and characteristics of the study samples (n=150)

**Variable**	**Total (n=150)** **No. (%)**
Age group (years)	
20-29	29 (19.9)
30-39	32 (21.3)
40-49	56 (36.8)
50-59	31 (20.7)
>59	2 (1.3)
Education status	
Illiterate	6 (4)
Elementary	67 (44.7)
Diplomma	44 (29.3)
High school	34 (22)
Income of family (Rials)	
<5000,000	93 (65.5)
5000,000-10,000,000	41 (28.9)
>10,000,000	8 (5.6)
Residency	
City	114 (75.5)
Rural–village	36 (24.5)
Occupation	
Employed	20 (13.4)
Housewife	130 (86.6)
Hypertension	
Yes	52 (34.7)
No	98 (65.4)
Type of treatment	
Oral hypoglycemic agent	138 (92)
Insulin	9 (6.0)
Oral hypoglycemic agent+Insulin	2 (1.3)
Only diet+exercise	1 (0.7)

In these patients, among the complications of DM, diabetic neuropathy was the most frequent (57.3%), followed by diabetic nephropathy (25.3%), diabetic retinopathy (16%), and major cardiovascular problems (10.7%), respectively. 

The mean of the total score of Female Sexual Function Index (FSFI) was 22. Prevalence of SD in this sample was 78.7% (CI: 71.4-84.4). Among these, 58% (CI: 50.0-65.6) reported problems in lubrication, 50% (CI: 42.1-57.9) complained of decreased sexual desire, 50% (CI: 42.1-57.9) had problems with arousal, 47.3% (CI: 39.5-55.3) had dyspareunia, 32.7% (CI: 25.7-40.5) complained of orgasmic dysfunction and 42.7% (CI: 35.0-50.7) reported problems in sexual satisfaction. 

The results of the Hospital Anxiety and Depression Scale (HADS) for frequency of anxiety and depression were as below: 

3.3% (CI: 1.4-7.6) of the cases had no anxiety; mild anxiety was present in 11.3% (CI: 7.2-17.4). About 39.3% (CI: 31.9-47.3) had moderate anxiety and 46.0% (CI: 38.2-53.9) reported severe anxiety. About 41.3% of the cases had no depression; mild depression was present in 17.3%. Nearly 16.7% had moderate depression and 24.7% of the cases reported severe depression. With regard to the results of the HADS, 58.7% (CI: 50.7-66.2) of the patients had depression and 96.7% (CI: 92.4-98.6) complained of anxiety.

*Sexual Dysfunction (SD) and Demographics and Diabetes Mellitus (DM) Related Factors*

The results showed that there was no significant association between the presence of SD and age of the patients (42.68±10.4 vs. 39.65±8.8, P=0.13), BMI (31.1±4.7 vs. 30.0±3.8, P=0.20), HbA1c level (7.93±1.8 vs. 7.82±1.7, P=0.76), duration of DM (7.93±5.7 vs. 6.25±4.7, P=0.13) and the presence of hypertension (P=0.69), menopause (P=0.12), type of treatment (P=0.13) and type of oral hypoglycemic agents (P=0.97). No significant association was also found between SD and the status of the disease control and complications of DM, as well (table 2).

**Table 2:** The association between sexual dysfunction (SD) and complications of diabetes mellitus (DM)

**Sexual ** **dysfunction**	**Diabetic complication**	**Yes (number/percent)**	**No** **(number/percent)**	**Chi-square Test** **P value**
Diabetic neuropathy	Yes	72 (83.7%)	14 (16.3%)	0.06
	No	46 (71.9%)	18 (28.1%)	
Diabetic retinopathy	Yes	21 (87.5%)	3 (12.5%)	0.192
	No	97 (77.0%)	29 (23%)	
Diabetic nephropathy	Yes	30 (78.9%)	8 (21.1%)	0.580
	No	88 (78.6%)	24 (21.4%)	
Cardiovascular complications	Yes	13 (81.3%)	3 (18.8%)	0.542
	No	105 (74.4)	29 (21.6%)	

*Psychological Factors in Women with Type 2 DM*

About 58.7% of the women reported depressive symptoms and 96.7% reported anxiety, according to the results of the HADS questionnaire. Among diabetic women with sexual problems, 95.8% were suffering from anxiety too (11.9% mild, 41.5% moderate, 42.4% severe) though anxiety prevalence was not significantly different between the two groups with or without sexual dysfunction (P=0.24).

There was a significant difference in depression among those with and those without SD (P=0.00). There was no significant difference in anxiety among those with and those without SD (P=0.19). 

## Discussion

Sexual Dysfunction (SD) is an important, but often neglected, component of health care in patients with diabetes mellitus (DM) and most recent studies suggest that SD occurs in a great proportion of diabetic women.^2^^,^^15^^,^^16^^,^^27^^,^^28^ This survey studied SD among 150 type 2 diabetic women using the Female Sexual Function Index (FSFI). The findings indicated that the patients were greatly affected by SD (78.7%). Generally, high rates of SD in diabetic women are reported in different researches. A study in Iran on 50 married women with type 2 DM and 40 non-diabetic women in 2009 showed that DM significantly impairs sexual function.^16^ Another study performed in Iran on 200 patients (100 males and 100 females) with either type 1 or type 2 DM showed that SD prevalence was remarkably high among the mentioned cases.^15^ In this study, 82.5% of the patients of both genders reported that they have had experienced at least one type of SD.^16^ A study by Wallner about sexual functioning among 1291 diabetic and non-diabetic women in Boston, demonstrated that women with type 2 DM might experience similar sexual functioning to women without DM, but women with type 1 DM might report more SDs such as dyspareunia.^28^ Another study was performed to evaluate the prevalence and correlation of female sexual function in 595 women with type 2 DM by Esposito in 2010. The overall prevalence of female SD among the type 2 diabetic women was 53.4%.^29^

In our study, decreased libido was reported in 50% of the patients, arousal problems in 50%, problems in lubrication in 58%, problems with satisfaction in 42.5%, pain during intercourse in 47.3% and finally 32.7% had orgasmic problems. In a long-term research, entitled “epidemiology of diabetes interventions and complications” (EDIC) evaluating 625 female participants, it was determined that 35% of the women with type 1 DM met the criteria for female SD. The women with SD reported loss of libido (57%), orgasmic dysfunction (51%), problems in lubrication (47%), arousal dysfunction (38%) and pain during intercourse (21%).^30^ Another study, also demonstrated reduced vaginal lubrication in diabetic women.^27^ A review on female sexual disorders in women with DM showed that these women were at higher risks for developing SD than non-diabetic cases.^31^ Whether sexual desire is affected by diabetes remains controversial as some studies have shown a 20-78% decrease in desire in diabetic women (with the higher prevalence encountered in type 2 diabetes), while other studies have found no effect at all.^2^^,^^8^^,^^16^^,^^32^^,^^33^ The incidence of arousal problems in women with diabetes is also varied, depending on the type of diabetes and the definition of arousality, and varies from 14 to 75% to no effect at all.^2^^,^^8^^,^^16^^,^^32^^,^^33^ Regarding orgasm, as in our study, most studies have indicated problems in women with diabetes ranging from 10-84%.^8^^,^^16^ Finally, the risk of dyspareunia in women with diabetes varies from zero to 43%, with the higher prevalence being observed in type 2 diabetes.^34^ Despite the inconsistency that exists in the literature concerning domains of sexual function, it seems evident that the effect of diabetes on female sexuality is different and could affect all the domains of sexual function.

In this study, we did not find any significant relationships between sexual dysfunction and age group. Ziiaee et al. also reported the same results in Iran,^15^ but other studies, even from different countries have reported age as a determinant of SD in patients with DM.^3^^,^^15^^,^^29^^,^^35^ Fattemi et al. reported that age was negatively correlated with all domains of sexual functioning in women with type 2 DM.^16^ In this study, neither income nor education, BMI, HbA1c, diabetic status, duration of DM, hypertension and diabetic complications were correlated with SD in the diabetic women of the study population. Similarly, two studies by Muniyappa et al. and Doruk et al. indicated that none of the supposed factors except for depression predicted SD in diabetic women.^35^^,^^36^ Esposito et al., from Italy, have reported no association between HbA1c level , duration of type 2 DM, hypertension or cigarette smoking and female SD; however, age, metabolic syndrome and atherogenic dyslipidemia had a significant relationship with female SD.^29^ Enzlin et al. found no association between age, BMI, duration of type 1 DM, HbA1c level, use of medications, menopausal status or diabetic complications and female SD.^2^ On the other hand, Bitzer et al. recommended that a careful glycemic control in women with type 2 DM was fundamental for restoring normal sexual function.^9^

In this study, 65.3% of the patients had depression and 95.8% had anxiety, according to the Hospital Anxiety and Depression Scale (HADS) questionnaire. There was a significant correlation between depression and female SD. Depression is common in women with type 2 DM, which should be diagnosed and treated. One review found that the incidence of sexual problems in women with DM has been generally more associated with psychological factors than organic ones, especially coexisting depression.^31^ Consistently, Enzlin et al. demonstrated that SD in women with type 1 DM was related to depression.^2^ In a research by Esposito et al. on 595 women with type 2 DM, depression was found as an independent predictor of female SD.^29^ In another study on 625 females with type 1 DM, depression was the major predictor of female SD.^30^ Even minor episodes of depression can affect woman’s sexual desire. Poor diabetic control or diabetic complications may cause depressive episodes and thus sexual dysfunction in diabetic women.^34^ Since many of the causes of female sexual dysfunction do not have a strict medical origin, one has to be careful about characterizing woman’s sexual problems as organic, that can lead to diagnosis mistake and further complication in their management.

The small differences in the frequency of sexual dysfunction between our study and other studies could be due to differences in sample size and recruitment of the group (general practitioners versus outpatient gynecology clinic or endocrinology clinic) and the used methodology (questionnaire versus questionnaire combined with a semi-structured interview)

This study provided useful information about SD using standard instruments in a group of women with type 2 DM. The small sample size of the study did not allow us to detect any statistical significance of the relation between SD and age, duration of DM, hypertension, etc. We also did not control the study for the presence of depression as an independent factor leading to SD; however, depression is 2-fold more prevalent in diabetics compared with the general population and it should not be excluded. 

For future studies, a multivariate analysis is recommended in order to exclude the effects of covariates on the association of variables. In this study, SD in the patients was only diagnosed by the FSFI questionnaire, not by psychiatric clinical interview, according to DSM-IV-TR and not by detecting the presence of distress in the patients using different instruments for assessing SD result in different estimates of SD prevalence. The scales, such as HADS, were not designed to be a clinical diagnostic tool. Self-assessment scales are only valid for screening goals. For a definite diagnosis, we need psychiatric interview.

We believe that DM could influence female sexual function via both psychological (e.g. depression, anxiety) and physiological (neuropathy, vaginal dryness, etc.) factors. These physiological and psychological factors are interrelated. The current evidence indicates that psychosocial rather than organic factors are implicated in the pathogenesis of the sexual impairment in women with diabetes;^34^ however, to date, very few researchers have studied the sequence of these events in a longitudinal study. Further longitudinal studies are essential to explain the casual relationship and mechanisms of associations between type 2 DM and female SD; moreover, prospective studies are needed. Since this study was a cross-sectional, it could not establish the causal relationship between SD and DM in women. The cross-sectional design of the study does not allow us to make any causal interpretation of the observed associations between psychological variables and sexual dysfunction. Our results suggest that psychological and not diabetes related somatic factors are related to sexual dysfunction in women with diabetes.

In addition, we did not have any control group and were not able to compare the data from this research with those in which normal population of patients with type 1 DM were the population of the studies. On the other hand, to date, few published epidemiological researches have studied sexual behavior and SD in the normal population in Iran.^15^^,^^16^^,^^22^ Furthermore, it was not possible to make an exact comparison between the data obtained from this study and those emerged from researches carried out in other countries because of cultural, social, economic and moral variables which enormously affect sexual function.

## Conclusion

This study showed that the prevalence of sexual dysfunction (SD) is high in women with type 2 diabetes mellitus (DM) and glycemic control is not correlated with the frequency of the dysfunction in these patients; however, SD is especially highly prevalent among those with comorbidity of depression. Health care professionals dealing with diabetic patients in their daily practice should be aware of the possible presence of SD in female patients; however, sexual performance is an aspect often neglected in daily practice. Sexual anamnesis has to be a routine part of evaluation in females with type 2 DM. A casual conversation about her sexual life and its problems may develop into an uncomfortable discussion for both the physician and the patient. Personal taboos regarding sex, confidentiality issues, worries about potential humiliation, time constraints, even the doctor’s limited experience in the management of sexual problems, are a few of the factors that can impede the uncovering of possible sexual difficulties or disorders. Physicians should pay more attention to discuss SDs when evaluating women with type 2 DM. Doctors should be aware of the problem and address issues of sexuality when they examine diabetic women. They should be trained to use appropriate methods for evaluating women’s sexual function in order to identify pertinent pathology and refer them to the appropriate management. In order to appropriately deal with women’s sexual issues, it is important to prepare a secure environment in the clinic, to improve the intelligence of health care providers about sexual issues, as well as increasing the number of psychiatrists and sex therapists. Recognition and multidisciplinary management of SD would be of great benefits for diabetic women. This fact is of great importance that sexual function should not be neglected in health care practice and clinical evaluations. 
